# Are there clinically relevant prognostic factors in diffuse large B-cell lymphoma beyond International Prognostic Index?

**DOI:** 10.2478/raon-2025-0028

**Published:** 2025-12-16

**Authors:** Milica Miljkovic, Vita Setrajcic Dragos, Gorana Gasljevic, Srdjan Novakovic, Lucka Boltezar, Barbara Jezersek Novakovic

**Affiliations:** 1 Department of Medical Oncology, Institute of Oncology Ljubljana, Ljubljana, Slovenia; 2 Faculty of Medicine, University of Ljubljana, Ljubljana, Slovenia; 3 Department of Molecular Diagnostics, Institute of Oncology Ljubljana, Ljubljana, Slovenia; 4 Department of Pathology, Institute of Oncology Ljubljana, Ljubljana, Slovenia; 5 Faculty of Medicine, University of Maribor, Maribor, Slovenia

**Keywords:** diffuse large B-cell lymphoma, next generation sequencing, new genetic types, prognostic factors

## Abstract

**Background:**

Diffuse large B-cell lymphoma (DLBCL) has variable prognosis, with only 50 to 60% of patients cured by standard first line treatment. Identifying patients unlikely to benefit from standard first line therapy is therefore crucial. Schmitz’s study identified four molecular subtypes of DLBCL with differing prognoses: MCD, BN2, N1, and EZB, with BN2 and EZB showing more favorable outcomes. This study aimed to evaluate the effectiveness of the Archer FusionPlex Lymphoma Assay in identifying the newly defined genetic subtypes of DLBCL, while also exploring the association between immunohistochemical (IHC) and next-generation sequencing (NGS) methods for classifying the cell of origin (COO) and assessing their predictive value for patient survival.

**Materials and methods:**

We classified 131 DLBCL patients using Hans algorithm into GCB (germinal center B-cell-like) and ABC (activated B-cell-like) subtypes, and with NGS applying Archer FusionPlex lymphoma assay into ABC, GCB, unclassified, and into Schmitz’s novel genetic subtypes. A mutational analysis of just 7 genes (*MYD88*^L265P^, *CD79B, EZH2, NOTCH1, NOTCH2, BCL2*, and *BCL6*) was used for genetic classification. Various statistical models were applied to assess survival differences between subtypes. Finally, STRATOS analysis was conducted to validate our preliminary statistical findings.

**Results:**

35.9% of patients were successfully classified into new genetic subtypes, with acceptable consistency between IHC and NGS method for COO determination. However, the new genetic subtype classification by NGS did not correlate with overall survival, nor did the COO classifications by IHC or NGS. The inclusion of these classifications also did not improve the predictive value of models compared to the basic model based on the International Prognostic Index (IPI) only.

**Conclusions:**

The Archer FusionPlex Lymphoma assay showed a somewhat lower detection rate of novel genetic subtypes compared to reports based on exome sequencing, yet identified novel genetic subtypes in over one-third of patients. However, an in-depth STRATOS statistical analysis did not confirm its predictive value for DLBCL prognosis, likely due to factors like patient selection and sample size limitations.

## Introduction

Diffuse large B-cell lymphoma (DLBCL) is the most common subtype of non-Hodgkin’s lymphomas (NHL), accounting for approximately 30% of all NHL.^1^ It is a heterogeneous disease in terms of clinical presentation, as well as in terms of its biological and pathological features. Diffuse large B-cell lymphoma, not otherwise specified (DLBCL, NOS), is the most common subtype, representing 80-85% of all cases.^1–3^ Still, even the DLBCL, NOS, is not a homogeneous entity and can be subdivided into several morphological, immunohistochemical and molecular subgroups.^1–3^

The addition of rituximab to standard chemotherapy CHOP (cyclophosphamide, doxorubicin, vincristine and prednisone) has substantially improved the outcomes of patients with the DLBCL, yet to a variable extent among different patients. Only between 50 to 60% of patients will be cured with R-CHOP, while up to 10% will be refractory to this treatment and another 30% will relapse after achieving their first complete remission.^4,5^ It is therefore important to identify upfront those patients who will not be cured with R-CHOP in order to tailor their individual first line treatment. One of the widely accepted robust prognostic tools to categorize the DLBCL patients in risk groups is the International Prognostic Index (IPI), introduced in the pre-rituximab era and validated also in the rituximab era.^6,7^

However, treatment outcomes can vary significantly, even within the same IPI risk group, and the IPI alone is not sufficient for the unequivocal identification of patients who may not be cured with R-CHOP. This outcome can be attributed to the important genetic and molecular heterogeneity of DLBCL, NOS, highlighting the need for the identification of additional prognostic markers.

Gene-expression profiling (GEP) studies have identified different molecular subtypes (“germinal center B-cell-like” – GCB, “activated B-cell-like” – ABC (non-GCB), and non-classified types of DLBCL) related to the cell of origin (COO), which are supposed to be of prognostic significance.^8–12^ Immunohistochemical (IHC) algorithms, such as the one proposed by Hans *et al*., have also been introduced as rapid and inexpensive alternatives to GEP that are readily available and have demonstrated reasonable concordance to gene expression profiling.^13^

Nonetheless, clinical interest extends to other prognostic markers, including the determinants of molecular heterogeneity in DLBCL, NOS, as indicated by the new molecular subtypes introduced by Schmitz *et al*., Chapuy *et al*., and other authors.^14–17^ The study of Schmitz *et al*. identified four molecular subtypes of DLBCL: MCD (based on the co-occurrence of *MYD88* and *CD79B* alterations), BN2 (based on *BCL6* fusions and *NOTCH2* mutation), N1 (based on *NOTCH1* mutation), and EZB (based on *EZH2* mutation and *BCL2* translocations), that were determined to be of prognostic significance.^14^ A more favorable prognosis has been predicted for BN2 and EZB in comparison to other two subtypes.^14^ Chapuy *et al*., on the other hand, identified five molecular subtypes showing certain overlapping with subtypes identified by Schmitz: C1 (resembling BN2), C2 (*TP53* mutation and *TP53BP1* alteration), C3 (resembling EZB), C4 (*RHOA* mutations, *TET2, ZFP36L1* alterations) and C5 (resembling MCD). If the genetic driver could not be identified, the DLBCL was categorized as C0.^15^ In the mentioned studies, in addition to genetic changes useful for genetic classification, gene expression signatures related to the tumor microenvironment, rearrangements of *BCL2* and *MYC* and other markers (such as *TP53* mutations, high proliferative activity, CD5, and CD30 expression) appear to play an important role in the prognostication process of high grade lymphomas.

To the best of our knowledge, there are currently no commercial gene panels available on the market specifically designed to define the genetic subtypes of DLBCL as determined in Schmitz’s or Chapuy’s classification.

Our retrospective study aimed to evaluate the effectiveness of the Archer FusionPlex Lymphoma Kit in identifying the new genetic subtypes of DLBCL as defined by Schmitz *et al*.^14^ Additionally, we examined the association between the IHC (Hans algorithm) and next generation sequencing (NGS) methods for classifying the COO and assessed their ability to predict survival.

## Patients and methods

### Patients

One hundred and thirty-one patients with DLBCL, NOS, were enrolled in this retrospective clinical study. The inclusion criteria were as followed: all patients were older than 18 years, were diagnosed with DLBCL at least 5 years prior to beginning of this study, and were (except of one patient) treated with R-CHOP/RCHOP-like therapy between 2011 and 2017 at the Institute of Oncology Ljubljana (OIL), Slovenia. This study included only patients with DLBCL, NOS, and excluded patients with testicular lymphoma, primary central nervous system lymphoma or plasmablastic lymphoma. Patients with HIV positive lymphomas were also excluded from this study. All clinical data were obtained from medical records available in hospital’s information system (patients’ age at diagnosis, clinical stage of the disease, data for IPI score, treatment protocols applied and number of treatment cycles, treatment outcomes - overall response rate [ORR], progression-free survival [PFS], and overall survival [OS]). Survival data were retrieved from the Cancer Registry of the Republic of Slovenia and survival status was censored for all patients on 20^th^ of June, 2023.

The study was approved by the National Medical Ethics Committee of the Republic of Slovenia (Approval Number 0120-103/2020/4) and by Institutional Review Board (Approval number KSOPKR-0012/2020) as well as the Institutional Medical Ethics Committee (Approval number EK-0120-103/2020/4). The requirement for individual informed consent was waived, as this was a retrospective database analysis. Additionally, the institutional informed consent form for treatment included permission to use patients’ data, materials, and/or test results for research purposes. The study was conducted according to the Declaration of Helsinki.

### Pathological examination

For all patients included in the study, paraffin blocks and corresponding hematoxylin and eosinstained slides were retrieved from the archive of Department of Pathology of the Institute of Oncology Ljubljana, and diagnoses were reviewed. The tissue microarrays (TMA) were constructed and IHC staining as well as interpretations were performed as already described by Boltezar *et al*.^18^ to classify patients according to the Hans algorithm into the GCB and ABC (non-GC) types.^13^ At the same time, material for genetic analysis was cut from each paraffine block. The review, TMA evaluations and the classification of patients according to the Hans algorithm, were performed by a skilled hematopathologist who was blinded to all clinical data.

### Next generation sequencing (NGS)

The Archer FusionPlex Lymphoma kit was selected for the NGS procedure due to its commercial availability and its targeting of 125 lymphoma-related genes, which we considered potentially advantageous for classifying samples into novel genetic subtypes.

RNA was isolated using the MagMAX™ FFPE DNA/RNA Ultra Kit (ThermoFisher, Waltham, MA, USA). A total of 250 ng of RNA was reverse transcribed into cDNA, and NGS was performed using the Archer FusionPlex Lymphoma Kit, following the manufacturer’s protocol (Invitae ArcherDX, San Francisco, CA, USA). The quality of the starting material was evaluated by assessing the quality of the cDNA synthesized from the RNA. For this purpose, the Archer PreSeq RNA QC assay was employed (InvitaeArcherDX, San Francisco, CA, USA). The library was quantified using the qPCR Library Quantification Kit (KAPA Biosystems, Wilmington, MA, USA) and sequenced on the MiSeqDx system (Illumina, San Diego, CA, USA). Data were analyzed using the Archer Analysis version 6.0.3.2. A genetic variant was considered true positive if the allele fraction was at least 10% and the coverage depth was at least 100x. Fusions were considered true positive if covered by five or more unique reads and represented over 10% of reads. Variants listed in the GnomAD database were excluded as germline. Only previously identified pathogenic variants were used for patient subgrouping. Variants were considered pathogenic if listed in Schmitz *et al*.^14^ Supplementary Table or the OncoKB database as oncogenic.^19^ Variants of uncertain significance and benign variants were excluded. *CD79B* gene amplification was considered true positive when its relative expression exceeded 8 on a 0-9 scale calculated by the Archer analysis software. Cases were classified by gene expression patterns into the ABC, GCB, and unclassified subgroups according to the COO classification. We used a simplified approach of mutational analysis of just 7 genes, as alterations in *MYD88*^L265P^, *CD79B, EZH2, NOTCH1, NOTCH2, BCL2*, and *BCL6* were employed for genetic classification into novel subtypes. This decision was based on literature data indicating that hallmark genetic alterations for each subtype include *MYD88* (66.2% prevalence) and *CD79B* (50.0% prevalence) for the MCD subtype; *EZH2* (44.7% prevalence) and/or *BCL2* (68.4% prevalence) for the EZB subtype; *BCL6* (72.8% prevalence) and/or *NOTCH2* (41.8% prevalence) for the BN2 subtype; and *NOTCH1* (100% prevalence) for the N1 subtype. Other alterations used by Chapuy and Schmitz to define specific genetic subtypes were mostly reported with a lower prevalence.^8^,^14^,^15^ Cases with *CD79B* and *MYD88* alterations were therefore classified as “MCD,” with *EZH*2 and/or *BCL2* as “EZB,” with *BCL6* and/or *NOTCH2* as “BN2” and with *NOTCH1* as “N1.” Remaining cases were genetically unclassified.^14^

### Statistical analysis

The median age, stage at the time of diagnosis, bone marrow infiltration, IPI score, number of treatment cycles, subtype by IHC (ABC and GCB), subtype by NGS (ABC and GCB and nonclassified) and new genetic subtypes according to Schmitz’s classification were determined. PFS was defined as the time from the end of first systemic treatment to disease progression or death from any cause for patients achieving partial (PR) and complete response (CR). OS was defined as the time from the date of diagnosis to the time of death from any cause. The PFS and OS were estimated using the Kaplan-Meier method and differences were compared using the log-rank test. The IPI score was used as a categorical variable: low (score of 0 or 1), low-intermediate (score 2), high-intermediate (score 3) and high-risk group (score of 4 or 5) for the purpose of survival analyses and as a numeric variable for STRATOS initiative analyses. P value ≤0.05 was considered to indicate a statistically significant difference. GraphPad Prism software 9.0.0 (GraphPadSoftware, Boston, MA, USA) was used for analyses. Additional statistical analysis was performed – namely the STRATOS analysis, to check our basic statistic. The analysis was run in R (v4.3.2) in RStudio (v2023.12.1+402) using the packages boot (v1.3.28.1), dplyr (v1.1.4), forcats (v1.0.0.), ggplot2 (v3.4.4), ggpubr (v0.6.0), gridExtra(v2.3), gtsummary(v1.7.2), Hmisc (v5.1.1.), kableExtra (v1.3.4), knitr (v1.45), lubridate (v1.9.3), readxl (1.4.3), pacman (v0.5.1), purr (v1.0.2), readr (v2.1.4), readaxl (v1.4.3), rio (v1.0.1), rms (v6.7.1), rsample (v1.2.0), stringr (v1.5.1), survival (v3.5.7) and survminer (v0.4.9), tibble (v3.2.1), tidyr (v1.3.0), tidyverse (v2.0.0), timeROC (v0.4), webshot (v0.5.5) and their dependencies. According to the guidelines of the STRATOS initiative, we also compared the calibration, discrimination, Brier scores and clinical utility of various tested Cox models in order to test our model more profoundly.^20–22^ Lacking an additional dataset for external validation, we validated our models using optimism-corrected internal validation with bootstrapping.

## Results

### Demographic data

A total of 131 patients with DLBCL were included in the study. The patients’ characteristics are summarized in Table 1. There was a slight female predominance, median age was 65 years (range 28-89). More than half of our group had stage IV disease (50.4%) and elevated serum lactate dehydrogenase level (LDH) (58.8%), while nearly half had B symptoms (49.6%). The highest percentage of patients were in the high-intermediate risk group (28.2% of patients), other three risk groups were quite evenly distributed. All patients except one (130 patients - 99.2%) received first line systemic treatment. Treatment regimen was R-CHOP (rituximab, cyclophosphamide, doxorubicin, vincristine and prednisolone) +/- middle dose of methotrexate (500 mg/m^2^) in 128 patients (97.7%). Two patients (1.5%) received R-COEP (rituximab, cyclophosphamide, vincristine, etoposide and prednisolone) therapy due to their cardiac conditions, while one patient underwent just palliative treatment (and was excluded from cohort analysis). The median followup time was 61 months (range 2–152 months).

**TABLE 1.** Patients’ characteristics

	N	%
Number of patients:	131	
Gender		
Male	59	45.0%
Female	72	55.0%
Age		
Age range	28–89	/
Median age	65	/
Stages (Ann Arbor):		
Stage I	19	14.5%
Stage II	25	19.0%
Stage III	21	16.0%
Stage IV	66	50.4%
Median stage:	4 (range 1–4)	
Other characteristics		
Bone marrow involvement	41	31.3%
Elevated LDH level	77	58.8%
B symptoms	65	49.6%
IPI group:		
Low risk	32	24.4%
Low-intermediate risk	31	23.7%
High-intermediate risk	37	28.2%
High risk	31	23.7%
Median IPI value:	3 (range 0-5)	
Treatment		
R-CHOP/R-CHOP like	128	97.7%
R-COEP	2	1.5%
Palliative care	1	0.8%
Treatment response	130	
CR	65	50.0%
PR	48	36.9%
SD	1	0.8%
PD	16	12.3%

1 CR = complete response; IPI = International Prognostic Index; PD =progressive disease; PR = partial response; R-CHOP = rituximab, cyclophosphamide, doxorubicin, vincristine and prednisone; R-CHOP like = R-CHOP +/- middle dose methotrexate; R-COEP = rituximab, cyclophosphamide, etoposide, vincristine and prednisone; SD = stable disease

For each patient, response to first line treatment was defined as CR, PR, stable disease (SD) or progressive disease (PD) based on the revised criteria of Cheson *et al*.^23^ The ORR for the entire group was 86.9% (with the CR at 50% and the PR at 36.9%).

### Classification of diffuse large B-cell lymphoma (DLBCL) according to the cell of origin (COO)

According to the COO determined by IHC method, 54 patients (41.2%) were classified as ABC subtype and 77 patients (58.8%) as GCB subtype. According to the NGS, 42 patients (32.0%) were classified as ABC subtype and 62 patients (47.3%) as GCB subtype, while 12 patients (9.2%) remained unclassified, and for 15 patients (11.5%) the NGS method could not provide a clear result (QC failed). NGS served as the reference method and concordance between the determined COO subtypes according to the IHC and the NGS method is presented in Table 2. The overall concordance between NGS determined COO and IHC determined COO in the whole study group was 61.8% (81 of total 131 patients).

**TABLE 2.** Concordance between the IHC and NGS method in determination of the COO. The reference method was NGS. A pairwise comparison between the results of both methods was performed for each patient. Each patient who was subclassified into the same (ABC or GCB) subtype by both NGS and IHC was considered concordant. Patients who were subclassified differently by IHC and NGS were considered discordant

	COO by NGS	COO by IHC	Concordance (%)
**ABC subtype**	42	31	73.8
**GCB subtype**	62	50	80.6

1 ABC = activated B-cell like; COO = cell of origin; GCB = germinal center B-cell like; IHC = immunohistochemical determination; NGS = next generation sequencing

### Genetic classification of diffuse large B-cell lymphoma (DLBCL) by next generation sequencing (NGS) according to the Schmitz’s classification

**FIGURE 1.**
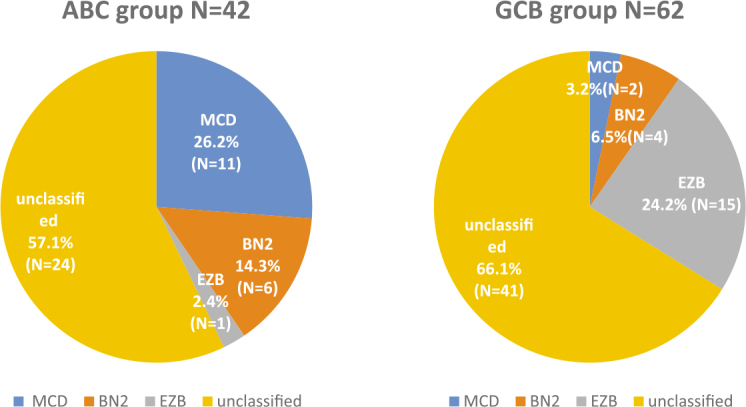
New genetic subtypes in the context of COO groups ABC and GCB, as determined by NGS (N1 group is not included in the Figure since there were no patients with this subtype). ABC = activated B-cell; COO = cell of origin; GCB = germinal center B-cell; NGS = next generation sequencing

**FIGURE 2.**
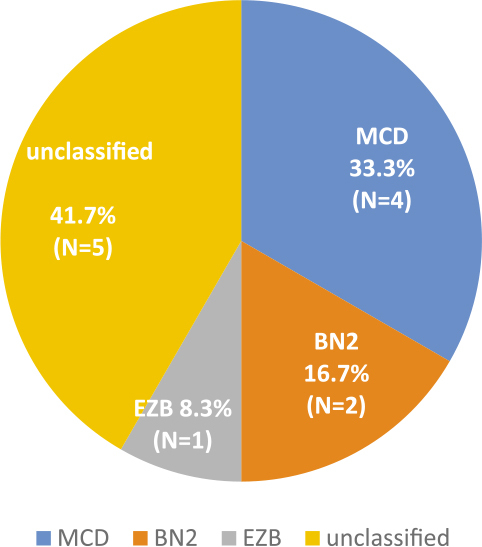
New genetic subtypes in the context of COO unclassified group, as determined by NGS (N1 group is not included in the Figure since there were no patients with this subtype). COO = cell of origin; NGS = next generation sequencing

Of the entire group of 131 patients, 47 patients (35.9%) were successfully categorized into one of the new genetic subtypes of DLBCL, 70 patients remained unclassified (53.4%), while 14 patients (10.7%) were categorized as QC failed. Among those 47 patients, 17 patients had MCD (13%), none had N1, 12 had BN2 (9.2%), and 18 patients had EZB (13.7%) subtype. New genetic subtypes in the context of COO groups ABC, GCB and unclassified group are presented in Figures 1 and 2.

When focusing only on the QC failed (as determined by NGS) group of 15 patients of the entire 131 patient cohort, 1 patient was categorized with the EZB subtype (6.7%). The remaining 14 patients remained unclassified to the new genetic subtypes.

### Overall response rate according to the immunohistochemical (IHC) and next generation sequencing (NGS) determination of cell of origin (COO) and NGS determination of new genetic subtypes

According to the IHC classification of COO into ABC and GCB subtype, the ORR for ABC subtype was 84.9 % and 88.3% for the GCB subtype. Based on the NGS classification of COO, the ORR for the ABC subtype was 82.9%, 88.7% for the GCB subtype and 91.7 % for the unclassified group. The ORR for MCD genetic subtype was 76.5%, for BN2 genetic subtype 91.7%, for EZB genetic subtype 94.4%, for unclassified group 87.1%, and for QC failed group 85.7%. There was no association between the ORR and classification to the abovementioned subgroups regarding COO by IHC, COO by NGS and classification to new genetic subtypes by NGS (p = 0.59, p = 0.76, and p = 0.80, respectively).

### Overall survival according to the immunohistochemical (IHC) and next generation sequencing (NGS) determination of cell of origin (COO) and NGS determination of new genetic subtypes, and according to the International Prognostic Index (IPI)

**FIGURE 3.**
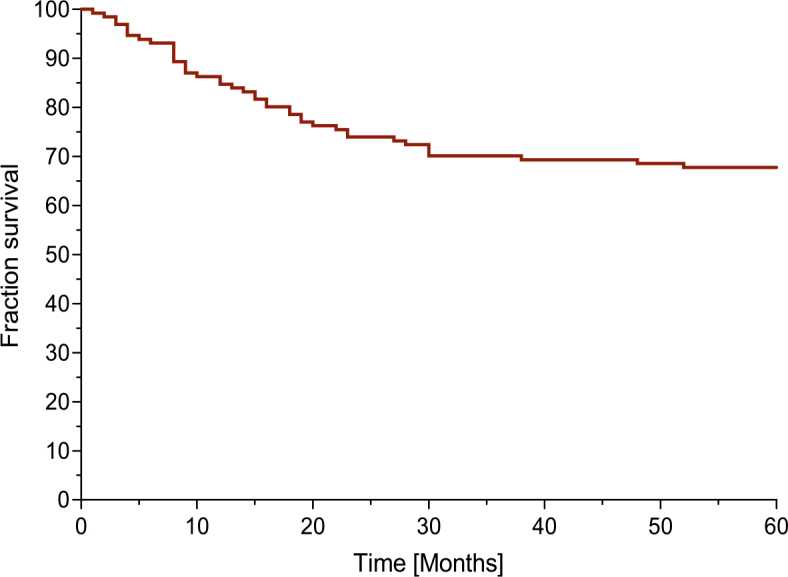
Overall survival (Kaplan-Meier) of all patients (N = 131).

**FIGURE 4.**
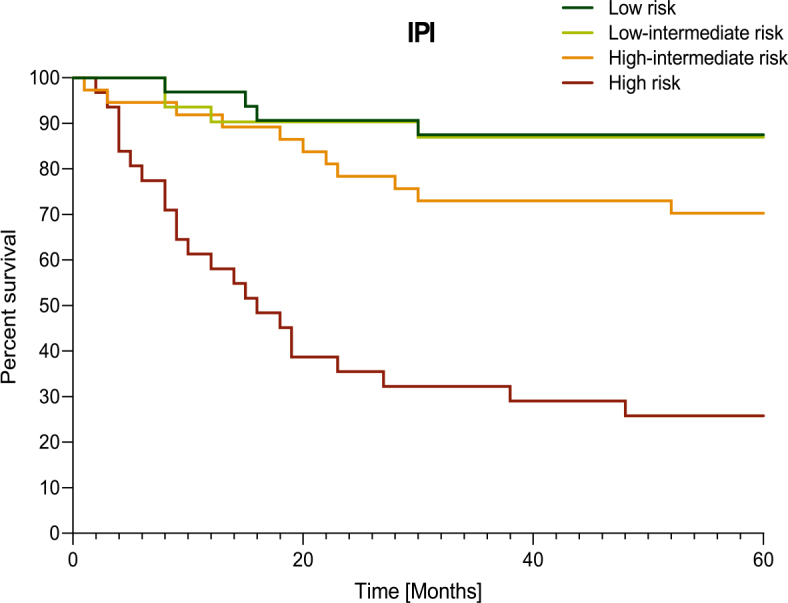
Overall survival (Kaplan-Meier) of patients according to International Prognostic Index (IPI) risk groups; (p < 0.0001).

Five-year overall survival (OS) of the entire group was 67.8% (Figure 3). According to the IHC classification of COO into ABC and GCB subtype, the 5-year OS for ABC subtype was 62.5% and 71.4% for GCB subtype. Survival was not significantly different between the two groups, (p = 0.27, HR = 1.36 [95% CI 0.76–2.42]) Supplementary Figure 1. Based on the NGS classification, the five-year OS for ABC subtype was 54.8% and 74.2% for GCB subtype, 64.2 % for the unclassified group and 80% for the QC failed group, and, again, there was no statistically significant difference between groups (p = 0.06) – Supplementary Figure 2. The 5-year OS for patients diagnosed with the new genetic subtypes according to the Schmitz’s classification was 66.7% for the BN2 subtype, *77.8%* for the EZB subtype, 64.7% for the MCD subtype, 78.6% in the QC failed group, and 63.9% for the unclassified group (p = 0.61) - Supplementary Figure 3. The 5-year OS was *87.5%* in low risk IPI group, *87.0%* in low-intermediate risk IPI group, 70.3% in high-intermediate risk IPI group and 25.8% in high risk IPI group (p < 0.0001, HR = 0.117 [95% CI 0.06–0.245]) (Figure 4).

### Cox models

We are not giving the PFS data as we consider them to be only of predictive and not of a prognostic significance. Even though we are aware of the potential influence of subsequent therapies to the OS, we chose to evaluate the prognostic significance of the three classifications that had been applied in the study – namely the COO by IHC classification, COO by NGS classification and the new genetic types by NGS classification. We used the Cox proportional model to investigate the association between the OS and the particular classification (COO by IHC, COO by NGS, and new genetic types by NGS, respectively) as well as the IPI score. The following Tables summarize the four models used - Supplementary Tables 1, 2 and 3 The base model uses only IPI as an independent variable and the three classifications available to IPI as a second independent variable. However, when combining IPI score and any of classifications (COO by IHC, COO by NGS, and new genetic types by NGS) into one model, none of the classifications had a significant prognostic impact on patients’ survival and only IPI remained prognostic for OS. So, regardless of the classification used, the survival of patients was not statistically significantly different between the ABC and GCB subtype (and unclassified subtype) or between the new genetic subtypes in our data set.

### STRATOS statistical analyses

The STRATOS initiative produced guidelines for comparing Cox models. For technical reasons, IPI is included as a numerical variable (i.e. the number of risk factors present). As shown previously, regardless of the model/classification used, only the IPI remains statistically significantly associated with survival.

Given that each individual classification (COO by IHC, COO by NGS, and new genetic types by NGS) was not associated with survival, we expected the subsequent analysis to show that inclusion of any one of the classifications into the model would not improve the quality of the model or its clinical usefulness.

### Discrimination and calibration

Corrected internal discriminations of the compared models at a fixed time point 5 years after diagnosis are given in Supplementary Table 4 and calibrations at a fixed time point 5 years after diagnosis are given in Supplementary Table 5. The addition of any classification did not improve the quality of the model, which was expected, given that none of them was significantly associated with overall survival.

### Overall quality

The Brier score and the scaled Brier score (Index of Prediction Accuracy, IPA) estimate the overall model quality. Brier score estimates the average difference between predicted and observed values at time t (and a lower value indicates a better model). IPA improves the interpretability of the score and estimates the reduction in Brier score when using a more complex model compared to the base model (a higher value therefore indicates a better model). The Brier score and IPA are given in Supplementary Table 6. We can conclude that the additional information about the classification does not improve the model quality (the addition of IPI compared to the null model improves the Brier score by 20%, while the extended models (with COO by IHC, COO by NGS and new genetic subtypes by NGS) improve it by 17% to 19%).

Calibration, discrimination and overall quality were assessed using bootstrap (B = 1000) and optimism corrected internal validation, since an additional data source for external validation was not available. As expected, we demonstrated that the addition of any one of the available classifications did not improve the performance of the base model that only included the IPI score.

### Clinical usefulness

Discrimination and calibration are necessary to assess the model quality or to compare different models, but they are not enough to evaluate their clinical usefulness. When an additional marker is available (in this case the subtype classification, whether histological or genetic), the evaluation of clinical usefulness should tell us whether the use of the additional marker leads to improved clinical decision making. We assessed the clinical usefulness of the extended models (with COO by IHC, COO by NGS and new genetic subtypes by NGS) compared to the base model at various possible risk thresholds. We have shown that the extended models do not improve the net benefit to patients when used in clinical decision making, regardless of the threshold or extended model chosen (Supplementary Figure 4, Supplementary Table 7).

### Discussion

DLBCL is highly heterogeneous in its genetic characteristics, making accurate sub-classification of patients based on tumor genetic changes essential for optimal treatment approaches. The classifications proposed by Schmitz and Chapuy are currently the most effective in grouping patients according to disease prognosis and treatment outcomes.^14,15^ However, no commercial tool (gene panel) is at present available that could classify patients into the genetic groups proposed by these authors. Therefore, the aim of our study was to evaluate the utility of the Archer FusionPlex Lymphoma Kit in classifying DLBCL into the groups outlined by Schmitz *et al*.^14^ Furthermore, we specifically assessed the impact of IHC classification of COO, NGS classification of COO, and NGS classification of new genetic subtypes based on Schmitz’s proposal on overall survival in patients with diffuse large B-cell lymphomas.

In fact, similar approaches for the genetic categorization of lymphomas have been used by other authors. Crotty *et al*. conducted a smaller study involving 41 DLBCL patients using the Archer FusionPlex Lymphoma platform to analyze a panel of 125 lymphoma-related genes and evaluate its concordance with the IHC Hans algorithm, observing an 80.5% concordance for COO.^24^ Another group, led by Scott, used the Lymph2Cx assay, to test 20 genes and compare it to GEP by NanoString to define the COO. This assay provided concordant COO definitions in 96% of their cases.^25^ Multivariate analyses in their report showed that COO defined by Lymph2Cx was independently prognostic of survival, regardless of the IPI score.^25^

However, in the present study, the overall concordance between NGS determined COO and IHC determined COO was lower, at only 61.8%, likely because we did not exclude the QC-failed samples and on account of the NGS determined unclassified group. Specifically, we observed a 73.8% concordance in the ABC group and 80.6% in the GCB group. In contrast, the study by Crotty *et al*. included far fewer patients (41) compared to ours (131), and while the proportions of ABC, GCB, and unclassified subgroups in Crotty’s study were evenly distributed, similar to our findings, they did not report any failed results.^24^

Regarding Schmitz’s proposed classification of the new genetic subtypes of DLBCL, we were able to subclassify 35.9% of our patients using the Archer FusionPlex Lymphoma kit. This represents a lower proportion of classified cases compared to Schmitz’s study, where 46.6% of patients were categorized into the new genetic subtypes.^14^ Considering the fact that Schmitz’s study performed exome and transcriptome sequencing, while we conducted a limited panel of RNA sequencing, the difference in the proportion of successfully classified cases is relatively small. Our cohort included a larger number of samples with mutations that were not helpful to classify correctly patients into subgroups, suggested by Schmitz. The clinical impact of these mutations is, nevertheless, still unknown. Still, in other studies using a classification similar to Schmitz’s, the proportion of successfully classified patients was also less than 100%. Lacey performed a whole-exome sequencing on tumor samples of 928 patients (including primary central nervous system lymphomas and plasmablastic lymphomas), in a panhematological malignancy panel of 293 genes, and found some overlapping groups over Schmitz’s and Chapuy’s classification. They identified 5 genomic clusters and had a 27% rate of “unclassified cases”.^16^ Wright and his colleagues created the LymphGen algorithm that provided a probabilistic classification of the tumor from an individual patient into a genetic subtype and with a similar methodology as Schmitz they managed to subclassify 63.1% of patients.^17^

A closer comparison of our results with those of other authors indicates that with our simplified approach, we detected a relatively low proportion of the EZB subtype – namely, 21.8% in Schmitz’s study, 18.9% in Lacey’s study, and only 13.7% in our study.^14,16^ The EZB subgroup is primarily included within the GCB cases. Since the GCB subgroup is more prevalent in our study (58.8% of GCB by IHC and 47.3% by NGS) compared to Schmitz’s study (28.2% of GCB cases), the relatively low EZB detection rate in our study remains unexplained.^14^

Schmitz *et al*. reported the predicted 5-year overall survival rates for the MCD, BN2 and EZB subtypes of 26%, 65%, and 68%, respectively, while in our study they were numerically superior - 64.7%, 66.7%, and 77.8%.^14^ Lacey’s genomic cluster MYD88, which overlaps with the MCD subtype, showed a 5-year OS of 62.8% in R-CHOP treated population.^16^ Their MYD88 group included testicular and primary CNS lymphomas, while in our study those patients were not included. Lacey’s BCL-2 cluster that overlaps with EZB subtype, and whose 5-year OS of 69.5% adjusts with the one reported in Schmitz’s study, was, compared to the EZB survival of our group, inferior.^16^ But, as stated previously, the EZB group was relatively weakly represented in our study in comparison with other studies.

Furthermore, Schmitz’s study investigated the survival data of only 119 patients (treated with R-CHOP/CHOP like chemotherapy) diagnosed with new genetic subtypes out of all 257 patients classified into novel subgroups, so their survival data deficiently cover only half of the new genetic subtypes’ population.^14^ To the contrary, our study reports survival data of all included patients. In Lacey’s study, only two thirds of patients were treated with R-CHOP, however, they reported results of survival for patients treated with R-CHOP separately.^16^ In some of the studies, genetic subclassification essentially had a prognostic impact on survival of their patients.^14,16,17^ Finally, Zhang *et al*. conducted a randomized phase II trial of addition of a targeted therapy to R-CHOP in patients with DLBCL, driven after the first cycle of R-CHOP by newly determined genetic subtypes. Their study was not powered to show survival differences, but it did meet its primary endpoint by achieving higher complete response rates with novel therapeutic approaches. This indicates that the spectrum of possible future decisions in choosing of an optimal first line therapy might have to be based on gene expression analyses.^26^

The classification of the new genetic types by NGS used in our study, however, was not associated with overall survival, as were also not the other two classifications of COO determined by IHC or by NGS. Similarly, the inclusion of any of the three classifications (COO by IHC, COO by NGS and new genetic types by NGS according to Schmitz’s proposal) improved neither the calibration and the discrimination nor the clinical utility of the tested models, when compared to the basic model including only IPI values.

One of the strengths of our study is the use of advanced statistical methods,^20,21,22^ as well as the thorough histopathological evaluation of all samples by a skilled hematopathologist who was blinded to the clinical data. The STRATOS analysis of Cox regression, a novel and advanced statistical method to analyse the potential differences between classifications, has, to the best of our knowledge, never been done in the setting of the DLBCL. This advanced methodology disclosed no difference in survival regardless of the classification (COO by IHC, COO by NGS, and new genetic types by NGS) used. Based on the data and subanalysis of this study, the only factor with valid prognostic significance for overall survival was IPI, which remained significant regardless of the classification method applied (COO by IHC, COO by NGS, or new genetic subtypes by NGS per Schmitz’s proposal). Schmitz *et al*. and Chapuy *et al*. also showed IPI’s prognostic significance for overall survival in a multivariate model.^14,15^ However, their studies reported also significantly different survival outcomes based on COO subtypes, which was not confirmed in our study. Additionally, the prognostic value of COO subtyping has been questioned in a Hungarian study of 247 DLBCL patients, where the COO subtype failed to predict prognosis.^27^ Thus, the prognostic impact of COO subtypes appears more complex than initially suggested by Hans *et al*.^13^

The disadvantages of this study are its retrospective nature, and when compared to the Schmitz’s, Chapuy’s and Lacey’s study, a smaller number of patients included (they included 574, 304 and 928 patients, respectively).^14–16^ Yet, the number is still higher than in Crotty’s and Scott’s study.^24,25^ Another limitation of this study is the relatively high number of samples that failed quality control (QC failed category). The sequencing quality control likely failed for several reasons. The most common issue was the contamination of sample with DNA, as indicated by an imbalanced ratio of RNA to DNA reads. In a few cases, the final library concentration was low, leading to insufficient coverage for meaningful analysis. On the other hand, the limited number of just 7 genes analyzed in our study can be either regarded as a limitation of the study on classification capabilities into novel genetic subtypes due to the reduced impact of alterations in other genes (mutated in lymphoma) or as the strength of the study by offering a simplified approach to this classification in clinical practice.

## Conclusions

The Archer FusionPlex Lymphoma assay tested in our study showed a somewhat lower detection rate of novel genetic subtypes compared to reports based on exome sequencing. An in-depth statistical analysis of patients’ survival across the groups defined by our approach did not confirm its value in predicting outcome of DLBCL patients. However, the difference in proportion of successfully categorized patients within novel genetic subgroups, as proposed by Schmitz *et al*., with Archer’s FusionPlex Lymphoma assay compared to exome sequencing was relatively small, making our simplified approach to classifying of DLBCL patients potentially useful in everyday practice.

## Supplementary Material

Supplementary Material Details
