# Supplementary material for: Are there clinically relevant prognostic factors in diffuse large B-cell lymphoma beyond International Prognostic Index?
Source: Radiol Oncol. 2025 Dec 16;59(4):607–16. doi: 10.2478/raon-2025-0028 (PMC12707449; doi:10.2478/raon-2025-0028)
Supplement: Supplementary file 1 — Supplementary Material Details [file raon-2025-0028_sm.pdf]

# Are there clinically relevant prognostic factors in diffuse large B-cell lymphoma beyond International Prognostic Index?

Milica Miljkovic, Vita Setrajcic Dragos, Gorana Gasljevic, Srdjan Novakovic, Lucka Boltezar, Barbara Jezersek Novakovic

doi: 10.2478/raon-2025-0028

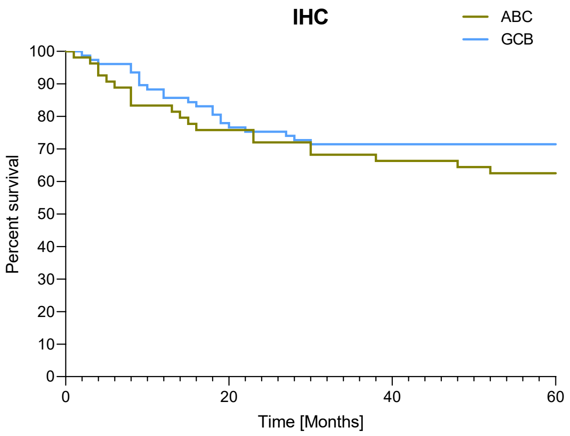


| Time [Months] | ABC | GCB |
|---|---|---|
| 60.000 | 62.532 | 71.429 |

**SUPPLEMENTARY FIGURE S1.** Overall survival (Kaplan-Meier) for ABC (N=54) and GCB subtype (N=77) as determined by IHC (immunohistochemical) determination; (p=0.27).

ABC = activated B-cell, GCB = germinal center B-cell

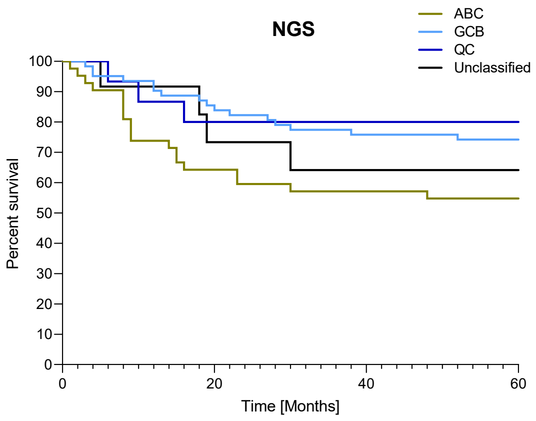


| Time [Months] | ABC | GCB | QC | Unclassified |
|---|---|---|---|---|
| 60.000 | 54.762 | 74.194 | 80.000 | 64.167 |

**SUPPLEMENTARY FIGURE S2.** Overall survival (Kaplan-Meier) according to the NGS classification; (p=0.06).

NGS = next generation sequencing, ABC = activated B-cell, GCB = germinal center B-cell, QC failed group = could not provide a clear result

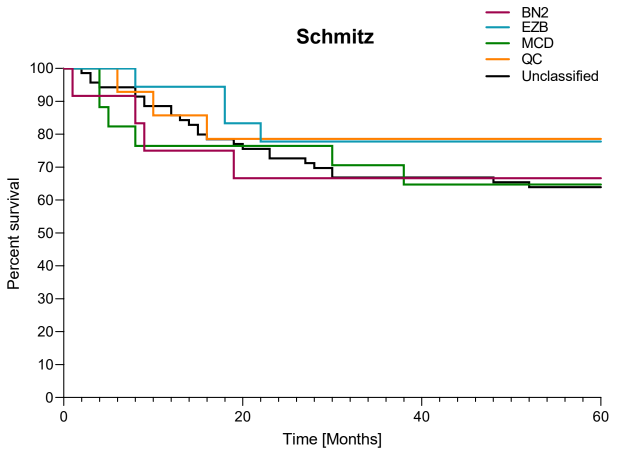


| Time [Months] | BN2 | EZB | MCD | QC | Unclassified |
|---|---|---|---|---|---|
| 60.000 | 66.667 | 77.778 | 64.706 | 78.571 | 63.941 |

**SUPPLEMENTARY FIGURE S3.** Overall survival (Kaplan-Meier) of new genetic subtypes determined by NGS according to the Schmitz's classification. (p=0.61).

NGS = next generation sequencing

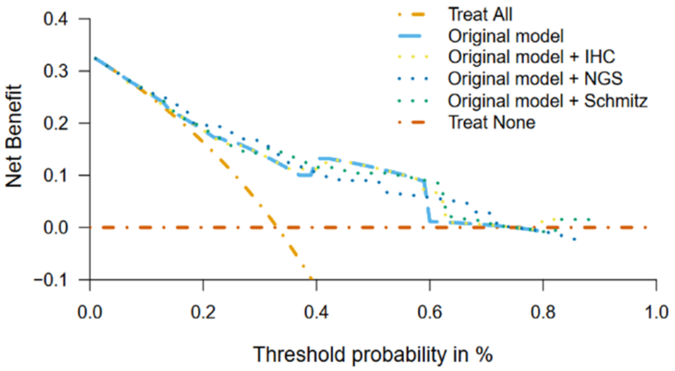


**SUPPLEMENTARY FIGURE S4.** Net benefit at different threshold probabilities for different ways of deciding which patients require further treatment.

IHC = immunohistochemical classification, NGS = next generation sequencing classification

**SUPPLEMENTARY TABLE S1.** Cox proportional model: association between OS, IPI score and IHC determination of COO

| | P value | HR | 95% CI |
|---|---|---|---|
| COO by IHC | 0.300 | | |
| IPI | **<0.001** | 1.873 | 1.48 - 2.369 |
| Lin | 0.000 | | |
| Quad | 0.722 | | |
| Cub | 0.239 | | |

COO = cell of origin, IHC = immunohistochemical determination, HR = hazard ratio, CI = confidence interval, Lin = linear function, Quad = quadratic function, Cub = cubic function

**SUPPLEMENTARY TABLE S2.** Cox proportional model: association between OS, IPI score and NGS determination of COO

| | P value | HR | 95% CI |
|---|---|---|---|
| COO by NGS | 0.053 | | |
| IPI | **<0.001** | 1.813 | 1.436 - 2.291 |
| Lin | 0.000 | | |
| Quad | 0.616 | | |
| Cub | 0.259 | | |

COO = cell of origin, NGS = next generation sequencing, HR = hazard ratio, CI = confidence interval, Lin = linear function, Quad = quadratic function, Cub = cubic function

**SUPPLEMENTARY TABLE S3.** Cox proportional model: association between OS, IPI score and new genetic types classification by NGS

| | P value | HR | 95% CI |
|---|---|---|---|
| New genetic types by NGS | 0.356 | | |
| IPI | **<0.001** | 1.857 | 1.483 - 2.326 |
| Lin | 0.000 | | |
| Quad | 0.784 | | |
| Cub | 0.327 | | |

NGS = next generation sequencing), HR = hazard ratio, CI = confidence interval, Lin = linear function, Quad = quadratic function, Cub = cubic function

**SUPPLEMENTARY TABLE S4.** Corrected internal discrimination 5 years after diagnosis

| | Uno AUC |
|---|---|
| Internal | 0.77 |
| Internal + COO by IHC | 0.77 |
| Internal + COO by NGS | 0.75 |
| Internal + new genetic subtypes by NGS | 0.75 |

COO = cell of origin, IHC = immunohistochemical determination, NGS = next generation sequencing, AUC = area under the curve

**SUPPLEMENTARY TABLE S5.** Calibration (values close to 1 represent good calibration and values close to 0.5 represent poor calibration)

| | Mean t | Mean interval | Weak t | Weak interval |
|---|---|---|---|---|
| No classification | | | | |
| Fixed internal | 0.97 | 1.00 | 1.03 | 1.03 |
| COO by IHC | | | | |
| Fixed internal | 0.97 | 0.99 | 0.91 | 0.92 |
| COO by NGS | | | | |
| Fixed internal | 0.98 | 0.98 | 0.91 | 0.92 |
| New genetic subtypes by NGS | | | | |
| Fixed internal | 0.98 | 0.97 | 0.87 | 0.88 |

COO = cell of origin, IHC = immunohistochemical determination, NGS = next generation sequencing

**SUPPLEMENTARY TABLE S6.** Brier score and IPA (Index of Prediction Accuracy)

| | Brier | IPA |
|---|---|---|
| Corr. internal | 0.18 | 0.20 |
| Corr. internal + COO by IHC | 0.18 | 0.19 |
| Corr. internal + COO by NGS | 0.18 | 0.18 |
| Corr. internal + new genetic types by NGS | 0.18 | 0.17 |

COO = cell of origin, IHC = immunohistochemical determination, NGS = next generation sequencing

**SUPPLEMENTARY TABLE S7.** Net benefit to patients when using different models to decide on additional treatment

| Threshold | 0.110 | 0.250 | 0.500 | 0.800 |
|---|---|---|---|---|
| Net benefit - treat all | 0.248 | 0.108 | -0.338 | -2.346 |
| Net benefit - basic model with only IPI | 0.254 | 0.167 | 0.115 | -0.008 |
| Net benefit - extended model with COO by IHC | 0.254 | 0.167 | 0.115 | -0.008 |
| Net benefit - extended model with COO by NGS | 0.255 | 0.182 | 0.085 | -0.008 |
| Net benefit - extended model with new genetic types by NGS | 0.257 | 0.156 | 0.108 | -0.008 |

COO = cell of origin, IHC = immunohistochemical determination, NGS = next generation sequencing
